# A case of hypoparathyroidism, deafness, and renal dysplasia (HDR) syndrome with a novel frameshift variant in *GATA3*, p.W10Cfs40, lacks kidney malformation

**DOI:** 10.1002/ccr3.3186

**Published:** 2020-08-14

**Authors:** Haruka Kishi, Teruo Jojima, Takahiko Kogai, Toshie Iijima, Eriko Ohira, Dai Tanuma, Sachiyo Konno, Kanako Kato, Atsumi Kezuka, Kazumi Akimoto, Junko Sakumoto, Akira Hishinuma, Takuya Tomaru, Noriko Makita, Isao Usui, Yoshimasa Aso

**Affiliations:** ^1^ Department of Endocrinology and Metabolism Dokkyo Medical University Mibu, Tochigi Japan; ^2^ Department of Infection Control and Clinical Laboratory Medicine Dokkyo Medical University Mibu Shimotsuga, Tochigi Japan; ^3^ Division of Clinical Science Research Support Center Dokkyo Medical University Mibu Shimotsuga, Tochigi Japan; ^4^ Center of Medical Ultrasonics Dokkyo Medical University Mibu Shimotsuga, Tochigi Japan; ^5^ Department of Nephrology and Endocrinology The University of Tokyo Tokyo Japan

**Keywords:** GATA3, HDR syndrome, hypoparathyroidism, sensorineural deafness

## Abstract

Autosomal dominant hypoparathyroidism, deafness, and renal dysplasia (HDR) syndrome are typically diagnosed by manifestations of the three features with a positive family history. Our case carried a de novo variant in causative gene, *GATA3,* but presenting no renal dysplasia or family history. The phenotypic heterogeneity raises a caution for diagnosis.

## Graphical abstract

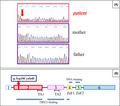

## 1 INTRODUCTION

We describe a patient diagnosed with hypoparathyroidism, deafness, and renal dysplasia (HDR) syndrome, but lacking one of the three features, renal disease. The detected germline variant in *GATA3*, p.W10Cfs, disrupting most of functional domains, could paradoxically cause the milder symptoms, compared to many other cases with a partial disruption.

HDR syndrome (OMIM #146255), also known as Barakat syndrome, is an autosomal dominant genetic disease caused by haplo‐insufficiency of *GATA3* located on chromosome of 10p14.^1^, ^2^ The clinical manifestation of the syndrome includes primary hypoparathyroidism, sensorineural hearing loss, renal dysplasia, cardiac malformation, and immune disorder. According to the ClinVar (https://www.ncbi.nlm.nih.gov/clinvar/), there are so far 14 pathogenic germline variants associated with HDR syndrome, including 3 missense variants, one nonsense variant, and 10 insertion‐deletion variants. Although HDR syndrome stands for hypoparathyroidism, deafness, and renal dysplasia, variable phenotypes of GATA3 variants have been reported,^3^ likely reflecting its wide tissue distribution, as well as the magnitude of alterations in GATA3. We recently experienced a case of HDR syndrome without renal dysplasia.

## 2 CASE PRESENTATION

A 24‐year‐old man was referred to our department for investigation of hypocalcemia. He was born from nonconsanguineous Japanese parents as a low birth weight immature baby of 1114 g with ptosis and joint contracture, although no neonatal asphyxia, cerebral dysgenesis, or multiple abnormality were described. His moderate bilateral sensorineural hearing loss was pointed out at the age of 4, resulting in requirement for a hearing aid. He often experienced facial spasm, tetany, as well as leg cramp, leading to falling down, at around the age 10, although those symptoms had been improved without intellectual disability during puberty. Since his tetany worsened again, he saw a primary physician, and pointed out hypocalcemia, (adjusted serum calcium, 6.1 mg/dL; reference interval 8.8‐10.1) and hyperphosphatemia (5.4 mg/dL; 2.7‐4.6). Oral administration of vitamin D and calcium lactate hydrate was started, resulting in serum calcium normalized and the symptoms improved. He had no family history of parathyroid diseases, tetany, deafness, kidney dysplasia, or other congenital anomalies.

**Figure 1:**
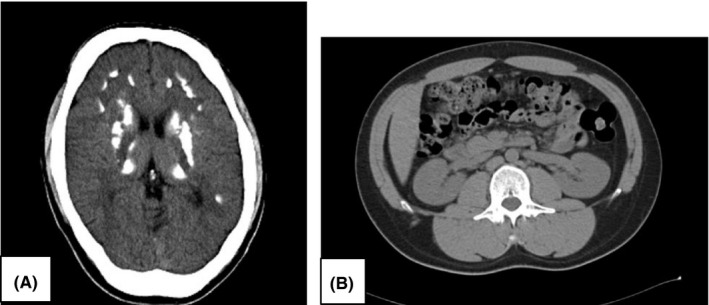
Radiographic imaging of brain, kidney. A, brain CT indicating multiple calcifications of basal nuclei. B, abdominal CT showing no obvious kidney malformation with transverse diameters of 90cm(left) and 93(right), respectively

Laboratory tests (Table 1) demonstrated marked reduction of intact PTH to 15 pg/mL (reference interval: 18.5‐88.0), but mild hypocalcemia with a serum calcium level of 8.3 mg/dL, and phosphorus within normal range at 4.9 mg/dL, due to the administrations of vitamin D and oral calcium supplement. Basal levels of other hormones were within the normal range. No evidence of renal dysfunction, proteinuria, hematuria, or urinary tract infection was obtained. Electrocardiography was negative for cardiac hypertrophy or ischemia. Neck ultrasound indicated no apparent nodule or abnormality in and around the thyroid glands. A brain CT showed multiple calcifications of basal nuclei (Figure 1A), while abdominal CT demonstrated no morphological abnormality of kidneys (Figure 1B).

**Table 1:** Laboratory data on admission

**Chemistry**		**Hematology**			**Blood gas analysis**	
AST	25 U/L	WBC	6600/µl		pH	7.328
ALT	25 U/L	RBC	53710^4^/µl		PCO_2_	40.3 mm Hg
T‐Bil	0.9 mg/dL	Hb	16.0g/dL		PO_2_	110 mm Hg
LDH	248 U/L	Hct	47.2%		HCO_3_‐	20.5 mmol/L
ALP	152 U/L	Plt	20.810^4^/µl		BE	‐4.7 mmol/L
ALP1	3%				AG	4.8 mmol/L
ALP2 + ALP3	91%	**Bone metabolic parameters**				
ALP5	6%	TRACP‐5b	781 mU/dL			
LAP	49U/L	Bone ALP	112 µg/L		**Urinalysis**	
gGTP	33 U/L	Deoxypyridinoline	2 nmol/mmol.Cre		pH	6.0
ChE	267 U/L	U‐NTx	10.6 nmolBCE/mmol.Cr		U‐glucose	‐mg/dL
TP	7.4 g/dL	calcitonin	15.7 pg/mL		U‐blood	‐
Alb	4.8 g/dL	intact‐PTH	15.6 pg/mL		BJ‐protein	‐
BUN	17 mg/dL	1,25(OH)_2_ Vit.D3	48.7 pg/mL		U‐ total protein	1.3 g/g.Cr
Cre	0.97 mg/dL	25(OH) Vit.D	22 pg/mL		Urinary NAG	2.6 IU/L
eGFR	81.5 mL/min					1.6 IU/g.Cr
UA	4.4 mg/dL	**Endocrinology**			Urinary β2‐MG	40 µg/g Cr
Na	140 mEq/L	F‐T4	1.1 ng/dL		Na	70 mEq/L
K	4.0 mEq/L	F‐T3	3.15 pg/mL		K	50 mEq/L
Cl	110 mEq/L	TSH	1.46 μU/mL		Cl	87 mEq/L
cCa	8.3 mg/dL	ACTH	23.1 pg/mL		Ca	4 mg/dL
IP	4.9 mg/dL	Cortisol	7.5 μg/dL		IP	57 mg/dL
Glucose	90 mg/dL	GH	0.4 ng/mL		UA	48 mg/dL
HbA1c	5.4%	LH	2.95 mU/mL		Cre	102 mg/dL
CRP	0.1 mg/dL	FSH	6.4 mU/mL		%TRP	88.94%
			PRL	6.8 ng/mL		

**Figure 2:**
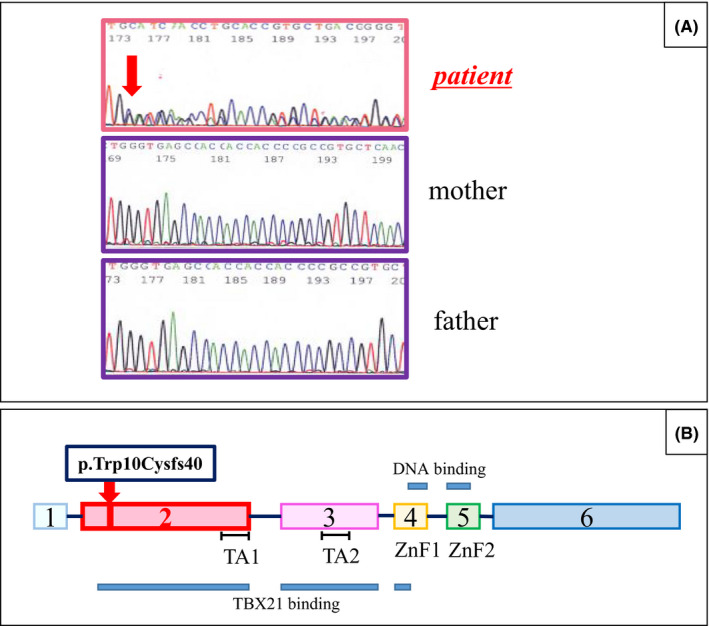
The germline pathogenic variant of *GATA3* detected in the present case. A, electropherograms around the codon 10. Upper panel indicates the heterozygous variant of *GATA3*, c.30‐49del‐insCACCGAGCTGCA, leading to p.Trp10Cysfs40, detected in the proband. Middle and lower panels, the genomic sequence from parents without the insertion‐deletion variant. B, locations of the variant and functional domains of *GATA3*. The pathogenic variant detected in this study leads to complete disruption of both the TBX21 binding site and the DNA binding domain. ZnF1 and 2, zinc finger domains, both required for efficient DNA binding. TA1 and 2, transactivation domains, in the TBX binding site

Differential diagnosis of early‐onset hypoparathyroidism includes several congenital diseases, DiGeorge syndrome, Kenny‐Caffey syndrome, and a genetic abnormality in *PTH* or *CaSR,* although deafness is almost always observed in HDR syndrome and more rarely in DiGeorge syndrome. To distinguish between HDR syndrome and DiGeorge syndrome, we evaluated classification of leukocytes by flow cytometry (Table 2), resulting in a decreased ratio of Th2/CD4 + T cells to 0.3% (0.32‐3.24), as well as an elevated ratio of Th1/Th2 to 76.2 (6.34‐29.67). These indicated a reduced differentiation of naive CD4 + cells into Th2 lymphocytes, consistent with HDR syndrome. FISH analysis did not reveal any microdeletion in the 22q11.2 region, indicating less possibility of DiGeorge syndrome. Those results encouraged us to perform a single gene analysis of *GATA3* with peripheral blood cells, even without renal anomaly, one of the three features of HDR syndrome. Informed consent for genetic studies was obtained from the patient and his family members, under the approval by the institutional ethical committee. The Sanger sequencing analysis demonstrated a heterozygous variant, c.30‐49del‐insCACCGAGCTGCA (Figure 2A, upper panel), leading to a frameshift variant, p.Trp10Cysfs40. Pedigree analysis indicated no symptoms of HDR syndrome, as well as no pathogenic variant in *GATA3*, in both parents (Figure 2A, middle and lower panels), indicating the insertion‐deletion variant detected in the proband was de novo.

**Table 2:** Laboratory data of circulating T‐cell subsets

		Reference range
CD4 + PD‐1+/CD4 + T cells (%)	2.18	2.00‐5.50
CD8 + PD‐1+/CD8 + T cells (%)	0.45	0.90‐5.92
CD4 + CD25+FoxP3+/CD4 + T cells (%)	0.93	1.44‐9.52
Th1 (IFNc + IL‐4‐)/CD4 + T cells (%)	19.1	3.9‐26.2
Th2 (IFNc‐IL‐4+)/CD4 + T cells (%)	0.3	0.32‐3.24
Th1/Th2 ratio	76.2	6.34‐29.67

To perform genetic testing in his parents, several genetic counseling sessions were required for their acceptance. Disclosing the results, however, much improved their mood and reduced their anxiety, without apparently affecting their relationship.

## 3 DISCUSSION

HDR syndrome is a rare autosomal dominant disease, caused by haplo‐insufficiency of *GATA3*, typically leading to the three major symptoms: hypoparathyroidism, sensorineural hearing loss, and renal dysplasia. Differential diagnosis of early‐onset hypoparathyroidism complicated with deafness includes HDR syndrome and DiGeorge syndrome. In the present case, low Th2 lymphocytes and absence of 22q11 abnormality, strongly suggested HDR syndrome. Genetic testing of *GATA3* indicated a frameshift variant. Although HDR syndrome is autosomal dominant, both of his parents did not carry the gene variant, showing the patient had a de novo deletion‐insertion variant.

About 60 percent of patients with the syndrome represent all of the three manifestations, while another 27 percent lack the kidney diseases,^1^, ^3^, ^4^ as observed in the present case. Renal dysplasia is often associated with a worse prognosis.^5^ GATA3 is abundantly expressed not only in the tissues associated with HDR syndrome, parathyroid, inner ear, and kidney, but also in lymphoid tissue, thyroid gland, pancreatic β‐cells, and gonads.^6^ Of those tissues, only three tissues, parathyroid, inner ear, and Th2 cells, were markedly affected in the present case.

*GATA3* haplo‐insufficiency is thought to be a major pathogenic mechanism of HDR syndrome. Dominant‐negative effects by a pathogenic variant, however, also play roles in some cases.^7^ There are so far two types of dominant‐negative variants in *GATA3,* with or without DNA‐binding activity. A classical dominant‐negative variant is KRR, which has an amino acid substitution between two zinc‐finger motifs.^8^ Such variants likely affect the function of unaffected GATA3 by occupying the GATA3 binding site. A following study has demonstrated that p.Cys321Ser exerts a dominant‐negative effect even without DNA binding, likely due to competitive interactions with other GATA3‐binding proteins.^7^

GATA3 regulates differentiation of T lymphocytes as a transcription factor, interacting with another transcription factor, TBX21, through the N‐terminal binding domain.^9^ According to the ClinVar (Table 3), a large number of pathogenic variants reported in *GATA3* are located in or around the DNA binding domain, downstream the TBX21 binding domain (1 to 257 a.a. according to the UniProt, https://www.uniprot.org/uniprot/P23771, see Figure 2B). Such variants will disrupt the DNA binding domain, but the TBX21 binding domain likely remains, leading to the dominant‐negative effects on the functionality of GATA3 derived from the other unaffected allele. The frameshift variant detected in the present case, located just 30 to 49 bases downstream from the ATG start codon, likely disrupted both the DNA‐binding domains and the TBX21 binding domain, so it would not retain the dominant‐negative activity, but represent an effect of haplo‐insufficiency. Lack of renal manifestation in the present case could be due to the disruption of TBX21 domain. Since a previous case with p.Met36fs was associated with kidney disease (Table 3), the N‐terminal portion between 10 and 36 could be important for the differential phenotype.

**Table 3:** Missense and insertion/deletion variants of *GATA3* judged as "pathogenic" by ClinVar (https://www.ncbi.nlm.nih.gov/clinvar/). Data were retrieved at 27 April 2020

Substitution		Phenotype			Country	Reference citation in ClinVar
Nucleotide	Amino acid	H	D	R		
c.108_109del	p.Met36fs	(+)	(+)	(+)	Italy	^4^
c.431del	p.Gly144fs	(+)	(+)	(+)	Spain	^10^
c.465_513del	p.Thr156fs	(+)	(+)	(+)	Germany	^2^
c.478del	p.Asp160fs	(‐) or (+)	(+)	(‐)	Taiwan	^11^
c.587del	p.Leu196fs	NA	NA	NA	NA	direct submission
c.646dup	p.His216fs	NA	NA	NA	NA	direct submission
c.681_682insACCACCCCATCAGCACTCACCCGCCCTACGTGCCC	p.Glu228fs	NA	NA	NA	Israel	direct submission
c.681dup	p.Glu228fs	NA	NA	(+)	NA	direct submission
c.823T > A	p.Trp275Arg	(+)	(+)	(+)	Japan	^5^
c.829C > T	p.Arg277Ter	(+)	(+)	(‐)	NA	^2^
c.924 + 2delinsGCTTACTTCCC	p.Ala309delins	(+)	(+)	(‐) or (+)	Taiwan	^11^
c.946_957del	p.Ser316_Asn319del	(+)	(+)	(+)	NA	^2^
c.1025G > A	p.Cys342Tyr	(+)	(+)	(‐)	Portugal	^12^
c.1051‐1G > C	p.Asn351_443Glydel	NA	NA	(+)	India	direct submission
c.1059A > T	p.Arg353Ser	(‐) or (+)	(+)	(‐)	Taiwan	^11^
c.1099C > T	p.Arg367Ter	(+)	(+)	(+)	Japan	^5^

Abbreviations: D, deafness; H, hypoparathyroidism; NA, not available; R, renal anomaly.

In conclusion, the novel *GATA3* frameshift variant, close to the start codon, represented relatively mild symptoms in HDR syndrome. The present case indicates the usefulness of genetic testing of *GATA3* for clinical diagnosis in patients with hypoparathyroidism complicated with deafness, even without renal dysplasia or positive family histories.

## CONFLICT OF INTEREST

The authors declare that there is no conflict of interest that could be perceived as prejudicing the impartiality of this case report.

## AUTHOR CONTRIBUTIONS

All authors have read and approved the manuscript. H.K, T.J, T.K, and T.I: wrote the manuscript. E.O, D.T, and K.K: diagnosed and treated the patient. S.K: performed ultrasound and interpreted the data. T.K, A.K, K.A, J.S, and A.H: carried out gene analysis and interpreted the genetic data. T.T, N.M, I.U, and Y.A: interpreted clinical data and participated in the first draft of the manuscript.

## PATIENT CONSENT

Written informed consent was obtained from the patient for publication including genetic data.
